# Next-Generation Sequencing Reveals Novel Homozygous Missense Variant c.934T > C in *POLR1C* Gene Causing Leukodystrophy and Hypomyelinating Disease

**DOI:** 10.3389/fped.2022.862722

**Published:** 2022-05-24

**Authors:** Muhammad Imran Naseer, Angham Abdulrahman Abdulkareem, Peter Natesan Pushparaj, Samah Saharti, Osama Y. Muthaffar

**Affiliations:** ^1^Center of Excellence in Genomic Medicine Research, King Abdulaziz University, Jeddah, Saudi Arabia; ^2^Department of Medical Laboratory Technology, Faculty of Applied Medical Sciences, King Abdulaziz University, Jeddah, Saudi Arabia; ^3^Department of Biochemistry, Faculty of Science, King Abdulaziz University, Jeddah, Saudi Arabia; ^4^Department of Pharmacology, Center for Transdisciplinary Research, Saveetha Dental College and Hospitals, Saveetha Institute of Medical and Technical Sciences, Chennai, India; ^5^Department of Pathology and Microbiology, King Abdulaziz University, Jeddah, Saudi Arabia; ^6^Department of Pediatrics, Faculty of Medicine, King Abdulaziz University, Jeddah, Saudi Arabia

**Keywords:** *POLR1C*, intellectual developmental disorder, leukodystrophy, hypomyelinating disease, WES, Saudi family

## Abstract

Leukodystrophies are a diverse group of genetically established disorders categorized by unusual white matter changes on brain imaging. Hypomyelinating leukodystrophies (HLDs) are a group of neurodevelopmental disorders that affect myelin sheath development in the brain. These disorders are categorized as developmental delay, spasticity, hypotonia, and intellectual disabilities. We describe a patient with developmental delay, cerebellar ataxia, spasticity, hypotonia, and intellectual disability from a healthy family member. Whole exome sequencing (WES) was performed to identify causative variants, which were further analyzed by bioinformatic analysis. WES was performed, and Sanger sequencing-based segregation analysis confirmed the presence of the homozygous missense variants of NM_203290.3 c.934T > C p.Ser312Pro of RNA polymerase I and III subunit C (*POLR1C)* gene in this patient and heterozygous variant in the unaffected carrier father and mother, supporting the pathogenicity and inheritance pattern of this variant. Furthermore, the variant identified by WES was validated in healthy controls (*n* = 100) using Sanger sequencing analysis. Finally, our study explained the important use of WES in disease diagnosis and provided further evidence that the variant in *the POLR1C* gene may play an important role in the development of hypomyelinating leukodystrophy in Saudi families.

## Introduction

In the recent years, numerous classification systems for hypomyelinating leukodystrophies (HDLs) have been developed using clinical manifestations, imaging findings, and organelle-specific disorders (1–3). HLDs have diverse clinical and genetic conditions, but ataxia, motor ability, and intellectual disability are the most prominent in most patients with HLDs (4–6). The prototype of HLD1 is Pelizaeus–Merzbacher disease [PMD (MIM: 312080)], due to the variations in the myelin protein proteolipid protein 1 [PLP1 (MIM: 300401]). Furthermore, eight patients with HDL-11 (HLD11; 616494) were identified as negative for mutations in the *POLR3A* and *POLR3B* genes, whereas 13 compound heterozygous or homozygous mutations were identified in the *POLR1C* gene by Thiffault et al. (7).

A homozygous c.221A > G mutation in exon 3 of the *POLR1C* gene in the Hungarian proband with HLD11 was identified, resulting in the substitution of a highly conserved protein residue Asn74Ser (6). Furthermore, a consanguineous Libyan parent had a boy with a homozygous c.95A > T mutation in exon 2 of the *POLR1C* gene with HLD11, resulting in a p.Asn32Ile substitution at a highly conserved amino acid residue (7). More than 15 genes linked to HLDs have been reported in the OMIM database (8).

One study that includes 252 individuals with Treacher Collins syndrome (TCS3; 248390) was done, and the three patients with compound heterozygous mutations in the *POLR1C* gene were identified (9). Another study reported a 4-bp deletion in intron 8 in the sister and her more severely affected brother at the splice donor site, where 922+3_922+6del was identified, which may result in the skipping of exon 8 by Splendore et al. (10). In another study, a compound heterozygous mutation in *the POLR1C* gene in childhood ataxia with leukodystrophy with a missense mutation (c.713A > G: p.Asp238Gly) frameshift mutation (c.698_699insAA: p.Tyr233fs) was reported by Han et al. (11).

Intellectual disability is a common and highly genetical heterogeneous disease. The prevalence of mental retardation (MR) is estimated from 8.7 to 36.8 per 1,000 (12), which can vary greatly across countries. MR phenotypes can present in various syndromes, such as leukodystrophy, which are inherited in an autosomal recessive manner. The diagnosis of leukodystrophy is often delayed because it is difficult to distinguish it from other diseases. Mutations in the genes encoding the components of *POLR1C* can result in leukodystrophy. Recently, novel *POLR1C* mutations were reported in two Korean siblings with ataxia and leukodystrophy (11). To date, 95 variants have been reported in *POLR1C* genes. Although leukodystrophy is usually inherited in an autosomal recessive manner, cases of autosomal dominant inheritance have also been reported (7). However, several disease-causing variants remain unknown. Here, we performed whole-exome sequencing (WES) to identify a variant in a patient with MR, developmental delay, cerebellar ataxia, spasticity, hypotonia, and intellectual disability from a non-affected family in Saudi Arabia.

## Materials and Methods

### Samples Collection and Ethical Approval

**FIGURE 1:**
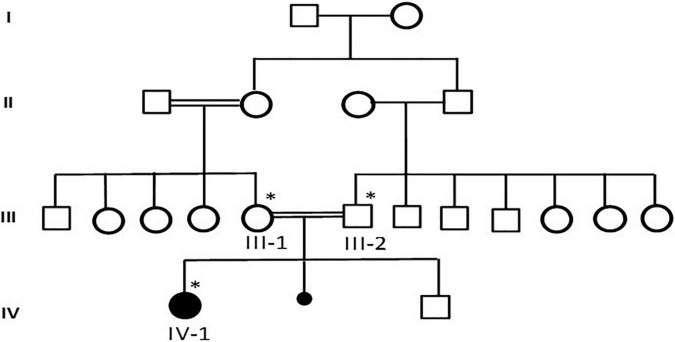
A detailed pedigree of family was drawn after information from the parents. The available samples were marked as symbol satiric. * represent the available sample for analysis.

This study was performed according to the established ethical protocol guidelines. Blood samples from the affected parents were obtained after signing written informed consent following the protocol in accordance with the Declaration of Helsinki. This study was approved by the local ethical committee of CEGMR, King Abdulaziz University (ethical approval number:013-CEGMR-02-ETH) and followed all the guidelines according to the Declaration of Helsinki 2013. DNA was extracted from the blood samples (Roche Life Science), as previously described (13). A detailed family pedigree was drawn after obtaining information from the family members, as shown in Figure 1. The DNA concentration was determined using NanoDrop™ 2000/2000c spectrophotometers from Thermo Fisher Scientific Waltham, Massachusetts, United States.

### Clinical Report of the Patient

**FIGURE 2:**
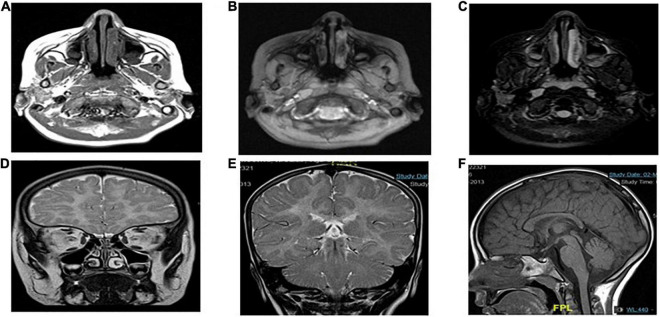
MRI showing the characteristics of *POLR1C* related leukodystrophy caused by POLR1C variant. Axial T2-weighted **(A,B,D,E)** and sagittal T1-weighted **(F)** images of case at the age of 4 years at the time of MRI.

Case presentation: The patient was a 7-year-old Saudi girl with a history of neuromuscular disease and normal neurodevelopment during infancy. At the age of 3 years, she started developing action tremor of her fingers, had difficulty in writing, showed early signs of motor dyspraxia, and developed amblyopia secondary to hypermetropia and astigmatism. However, there were no other neurological abnormalities without apparent facial abnormalities or microcephaly. No delay or abnormal order in presentation was noted. The deep tendon reflexes of the lower limbs had increased. She exhibited a staggering wide-based gait and was unable to stand on one leg for > 2 s. She presented with mild-to-moderate intellectual disability, hypotonia, ataxia, and unsteady gait, which required support, delayed speech, and delayed walking. There was no family history of any disease. Both parents and other family members were non-symptomatic (Figure 1). Due to the patient’s mental status, her physician ordered brain magnetic resonance imaging (MRI), which revealed that the white matter was predominantly involved (Figure 2).

### Magnetic Resonance Imaging

**FIGURE 3:**
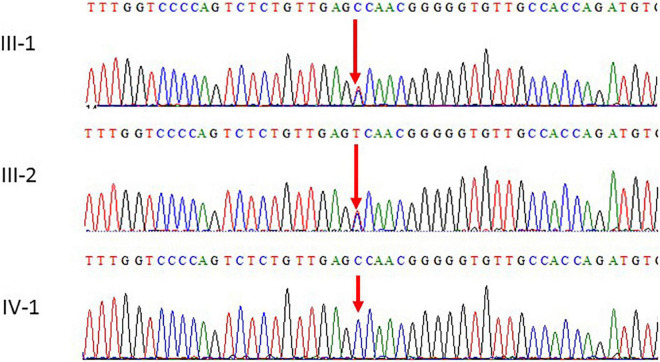
Chromatogram of Sanger sequence analysis III-1 and I-2 are the normal parents, while IV-1 are the affected member of the family showing a novel homozygous missense c.934T > C (p. S312P) of *POLR1C* gene.

Magnetic resonance imaging (MRI) scans of the patients were performed, and the findings were compatible with leukodystrophy. Metachromatic leukodystrophy and Pelizaeus–Merzbacher disease were more likely to be differentially diagnosed (Figure 3).

### Whole Exome Sequencing

To detect potential variants in the family, WES was performed using Illumina NextSeq 550 (High-Output v2 kit). The products were sequenced on an Illumina NextSeq instrument with 2 × 76 paired-end reads as previously described (14, 15). Quality control was maintained to achieve the highest quality of data on Illumina sequencing platforms, and it is important to create optimum cluster densities across every lane of every flow cell. This requires accurate quantitation of DNA library templates. Roche’s rapid library standard quantification solution and calculator were used to generate a standard curve of fluorescence readings and calculate the library sample concentration.

Therefore, we quantified the prepared libraries using qPCR, according to the Illumina qPCR Quantification Protocol Guide. All exomes were considered for sequence gain > 95% of bases covered for at least 15 reads throughout the whole exome. After WES, the FASTQ files were converted to BAM files, which were then converted to variant call format (vcf) files with a total of 103,265 variants. These variants were utilized to identify mutations that may lead to the disease based on novel/rare (MAF+0.01%) frequency, functional (predicted damage by Polyphen/SIFT), homozygous or heterozygous state, genomic position, pathogenicity, protein effect, and earlier associations with the disease-related phenotype. We used various filters and bioinformatic tools. All the reads obtained were aligned to the reference sequence of GRCh37. Variants were then filtered to identify disease-causing mutations in available public databases for allele frequencies < 5.0% in the Genome Aggregation Database (gnomAD),^1^ and frameshift, non-sense, and splice-site variants in disease-associated genes with a minor allele frequency ≤ 1.0% were observed in gnomAD. However, variants with minor allele frequencies ≤ 5.0% in gnomAD in a patient-specific phenotype-driven gene were also observed. The evidence for phenotype causality was then evaluated for each variant resulting from the filtering strategies above, and variants were classified based on the American College of Medical Genetics and American College of Pathologists (ACMG/AMP) criteria (16) with ClinGen rule specifications.^2^ Variants were reported according to human genome variation society (HGVS) nomenclature.^3^ In addition, *in silico* analyses were performed for missense variants to predict the effect of amino acid substitutions on the protein structure. Each candidate variant was evaluated using two online software programs: SIFT^4^ and PolyPhen-2 software.^5^ We followed the standard guidelines of the ACMG. *In silico* analysis of the structure and function of the identified variant was performed to check for deleterious effects and abnormalities that may be caused by mutations. We used software for *in silico* predictions, such as Mutation Tester,^6^ 1,000 Genomes database,^7^ PhyloP,^8^ PhyloP.

GERP++,^9^ SIFT,^10^ PhastCons,^11^ CADD,^12^ SiPhy, and Exome Aggregation Consortium.^13^ PROVEAN and MAPP are used for the identification of structure or function and evolutionary conservation of the protein.

### Sanger Sequencing

Mutations identified using WES were further validated by the Sanger sequencing analysis technique using the targeted primers of the reported variant in *the POLR1C* gene. Sequencing data files were obtained from the AB1 sequencing unit. The sequencing file was aligned with the reference sequence using the BioEdit software. We followed the guidelines to search for the variations in the National Center for Biotechnology Information (NCBI) SNP database. This variant was also not identified in 100 unrelated healthy individuals in the population. Both parents were heterozygous carriers and the proband had homozygous variants.

### Generation of a 3D Polymerase I and III Subunit C Homology Model

A homology model of the *POLR1C* protein was built using the SWISS-MODEL platform (17). The FASTA sequence of *POLR1C* was obtained from the UniProt Knowledgebase (UniProt ID: O15160), corresponding to a 346-amino acid transcript (Ensembl ID: ENSG00000171453.13) (18). A template search using BLAST and HHblits was performed against the SWISS-MODEL template library (SMTL version: 2021-02-16, last included PDB release: 2022-02-11), as previously described (19, 20). The target sequence was aligned to the primary amino acid sequence included in the SMTL, and an initial HHblit profile was generated (21, 22) and aligned with all profiles in the SMTL. In total, 99 templates were identified. The POLR1C homology model was constructed using (PDB ID:7ae1) based on the target and template alignment using ProMod3 (23).

### Mutation Analysis of Polymerase I and III Subunit C Using Missense 3D

The effects of substituting proline for serine on the *POLR1C* homology model were assessed using the missense 3D algorithm (19, 20, 24). However, buried proline insertion, secondary structure change, buried salt bridge break, buried charge change, buried glycine replacement, collision, buried hydrophilic residue insertion, buried charge insertion, buried charge replacement, buried H-bond break, cavity change, buried or exposed change, allowed phi/psi, disulfide bond break, and glycine in a bend were observed (19, 20).

## Results

### Magnetic Resonance Imaging

Magnetic resonance imaging showed diffuse T2 hyperintensities and T1 isointensities in the white matter, which indicates hypomyelination. T1 and T2 shortening in the optic radiation, ventrolateral thalamus, and dentate nucleus were noted, as typically observed in Pol III-related leukodystrophy 8; cerebellar atrophy or thinning of the corpus callosum was not evident (Figure 3E).

### Whole Exome Sequencing

Whole exome sequencing (WES) was performed, and the vcf file was obtained with a total of 103,265 variants. Different bioinformatic tools were used to determine the causative variant by applying different filters based on the pathogenicity, frequency, genomic position, quality, protein effect, and any associations with the diseased phenotype.

After further filtration and prioritization, one homozygous mutation in *the POLR1C* gene, including c.934T > C, p.(Ser312Pro) exon 9, was expected to be the disease-causing mutation in the patient. The mutation c.T934C led to amino acid changes from serine (Ser) to proline (Pro). The results of bioinformatic prediction by SIFT and PolyPhen2 confirmed that the amino acid substitution p.S312P in protein *POLR1C* was deleterious (score = 0) and damaging (score = 1), respectively. All the software programs used predicted that the identified variant was disease-causing, as shown in Table 1.

**TABLE 1:** Showing the details of *in silico* analysis done for this study.

S. no	Online tools	Pathogenicity score for mutation in *POLR1C* gene c.934T > C (p. S312P)
1	Exome Aggregation Consortium version 0.3.1	0.0
2	1,000 Genomes	0.0
3	Polyphen-2 (v2.2.2, released in Feb, 2013)	1.0
4	SIFT	1.00
5	MutationTaster	Disease causing
6	MutationAssessor 2.0	0.6
7	GERP++	14.50
8	PhyloP (phyloP46way_placental)	2.0
9	SiPhy 0.5	12.07
10	GenomAD	0.01

Subsequently, the peripheral blood specimens of the parents were sequenced using Sanger sequencing. Sanger sequencing confirmed that the novel homozygous missense c.934T > C mutation was inherited from the unaffected father and mother. The parents of the patients were unaffected by the clinical phenotype. The results of the segregation analysis in this family also supported the pathogenic role of the homozygous variant.

### Sanger Sequencing Analysis

**FIGURE 4:**
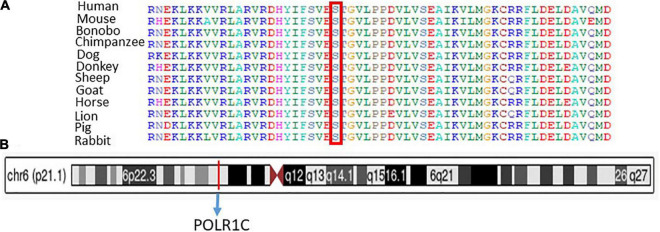
**(A)** Representation of different species alignment was done and highlighted the strong conservation of the amino acid variants at p.Ser312Pro. **(B)** Represent the chromosome, cytogenetic band, and genomic location of *PLOR1C* gene.

Sanger sequencing was used to validate the WES sequencing results by designing a set of primers for the observed variant. Sanger sequencing revealed a novel homozygous missense mutation c.934T > C, p.(Ser312Pro) exon 9, as shown in Figure 3. This variant was ruled out in 100 healthy control samples. The mutation c.T934C led to amino acid changes from Ser to Pro. The amino acid sequence alignment of different species highlighted the strong conservation of the variants at p.Ser312Pro, as shown in Figure 4A.

Chromosome 6: 43,509,241-43,511,229, cytogenetic band, genomic location of *the PLOR1C* gene are mentioned according to the UCSC Genome Browser on Human (GRCh38/hg38), as shown in Figure 4B.

### 3D Polymerase I and III Subunit C Homology Model and Mutational Analysis

**FIGURE 5:**
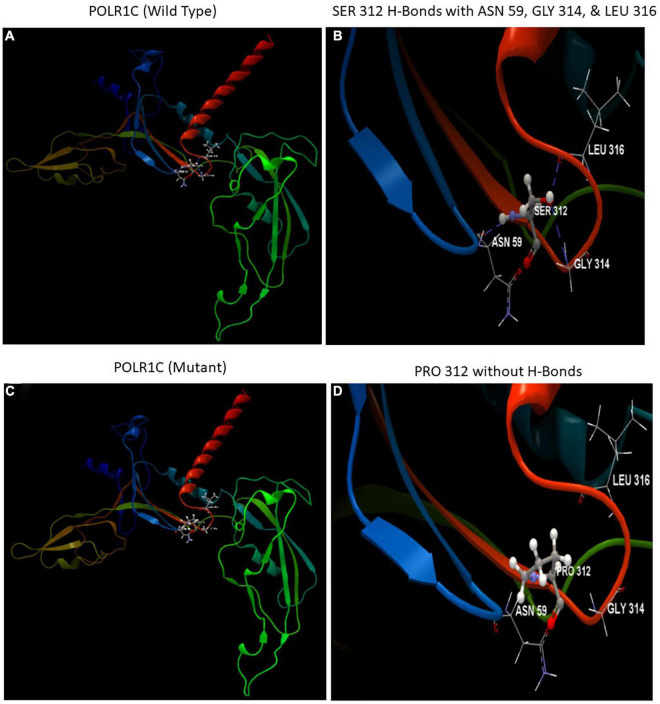
Illustration of the wild-type and mutant structures of POLR1C and the prediction for the position of p. Ser312Pro was generated using the homology modeling platform SWISS-MODEL. **(A)** The 3D structure of wild-type POLR1C and **(B)** hydrogen bonds (blue dotted lines) of SER 312 with ASN 59, GLY 314, and LEU 316 **(C)** The 3D structure of mutant POLR1C and **(D)** PRO 312 without hydrogen bonds. The amino acid SER at position 312 in wild-type POLR1C and PRO at position 312 in the POLR1C mutant are represented as ball and stick models.

The automated SWISS-MODEL homology modeling server was used to construct both wild-type and mutant protein structures for the location of p.Ser312Pro in the POLR1C gene (Figures 5A–D). Structural damage to the POLR1C protein is predicted because the substitution of proline at amino acid position 312 introduces a buried proline. In addition, the substitution of proline at position 312 in place of serine disrupted any H-bonds between the side chain and side chain and/or H-bonds between the side chain and backbone formed by a buried Ser residue (RSA 6.1%). However, in the POLR1C mutant protein, no disulfide bond disruption, no collision, buried hydrophilic residue, buried charge, altered secondary structure, buried charge change, false phi/psi, buried Gly, salt bridge disruption, altered cavity, buried or exposed switch, no replaced cis-Pro, and no Gly in a bend were observed (Figures 5A–D).

## Discussion

*The POLR1C* gene variant causing protein change p.S312P is classified as a variant of unknown significance according to the ACGME classification because of the following criteria. First, the (PM2 moderate) variant was not found in the gnomAD database. Second, (PP2 supporting), 37 out of 41 non-variant of uncertain significant (VUS) missense variants in POLR1C were pathogenic (90.2%), which is more than the threshold of 51.0%, and 47 out of 92 clinically reported variants in the gene *POLR1C* were pathogenic (51.1%), which is more than the threshold of 12.0%. Third (PP3 supporting), pathogenic computational prediction based on multiple automated algorithms was obtained.

Mutations in the *POLR1C* gene have been reported to cause leukodystrophy, hypomyelinating-11, and an autosomal recessive disease (OMIM# 616494). HDL 11 is a neurological disorder characterized by delayed psychomotor development and other neurological features associated with hypomyelination on brain imaging. In this study, we performed WES to identify variants in a patient diagnosed with MR from an unaffected family. WES provides a rational approach to concurrently screen for 14 candidate genes. After variant filtering, prioritization, and segregation analysis of family members, the suspected variants were highlighted. The potential causative variant was confirmed by Sanger sequencing. The missense variant c.934T > C, p. S312P, predicted by SIFT and PolyPhen2, showed a damaging effect on the protein function. Overall, the results of the bioinformatic analysis, segregation analyses, clinical phenotype coherence, and ciliopathy coherence supported that the homozygous variant of *POLR1C* was the causative variant of the patient, which was first reported. According to the guidelines of the American College of Medical Genetics and Genomics in 2015, the identified variants were classified as pathogenic with evidence levels of PM2, PM3, and PVS1 for p.Tyr233fs and PS3, PM1, PM2, PM3, and PP4 for p.Asp238Gly by Richards et al. (16).

*The POLR3* gene is involved in the synthesis of small non-coding RNAs that play vital roles in cell transcription and in RNA processing and translation (25). The POLR3 family and its members are the housekeeping genes that require strict regulations. A subset of leukodystrophies, named RNA polymerase III (Pol III)-related leukodystrophy or 4H (hypomyelination, hypodontia, and hypogonadotropic hypogonadism) leukodystrophy (MIM 607694, 614381)3, was found to be caused by biallelic pathogenic variants in genes that encode specific subunits of the enzyme Pol III, namely, POLR3A, POLR3B, POLR3K, and POLR1C.

Mutations in the POLR1C gene leading to the impairment of assembly and affecting nuclear import along with chromatin interaction of POLR3, which results in decreased binding to its target genes, reduce the transcription of tRNAs or other small non-coding RNAs that are central to the synthesis of proteins essential for CNS myelin development. POLR3A and POLR3B are the largest and second-largest subunits of RNA polymerase III (POLR3), respectively, which together comprise the catalytic core of the polymerase. POLR3A or POLR3B mutations cause the majority of POLR3-related leukodystrophy cases, with POLR1C mutations accounting for approximately 5% of cases. POLR1C is a common subunit of both RNA polymerase I (POLR1) and POLR3, which selectively alters the availability of both enzymes, resulting in two separate clinical conditions: Treacher Collins syndrome and POLR3-related leukodystrophy (11). However, the underlying pathophysiological mechanisms are not fully understood. As previously described as five distinct entities, these are now recognized as various clinical spectra of POLR3-related leukodystrophy (26).

The biochemical pathways related to POLR3 function are not yet fully known. Recent studies have been able to explain and characterize Pol III defects that may be produced by mutations that cause various overlapping rare diseases. However, the clear mechanisms linking these to hypomyelination and other phenotypic characteristics, such as hypogonadotropic hypogonadism and hypodontia, remain unknown (27–31). Further studies are required to elucidate the pathophysiology of POLR3-related disorders.

## Conclusion

In conclusion, we identified a homozygous missense variant of c.934T > C (p.S312P) in *the POLR1C* gene in this patient and a heterozygous variant in the unaffected carrier father and mother, further supporting the pathogenicity and inheritance pattern of this variant. Our study using the WES technique helped in disease diagnosis and elucidated that the variant in *the POLR1C* gene may play an important role in the development of leukodystrophy and hypomyelinating disease in the Saudi family.

## Data Availability Statement

The datasets for this article are not publicly available due to concerns regarding participant/patient anonymity. Requests to access the datasets should be directed to the corresponding author.

## Ethics Statement

Written informed consent was obtained from the individual(s), and minor(s)’ legal guardian/next of kin, for the publication of any potentially identifiable images or data included in this article. Written informed consent was obtained from the individual(s), and minor(s)’ legal guardian/next of kin for the publication of this case report.

## Author Contributions

MN conceived and designed the project. MN and AA performed the experiments, advised on the study design, and confirmed the results. MN, PP, and OM analyzed and interpreted the data. MN and OM provided and interpreted the patients’ phenotypic details. MN and SS prepared the manuscript. All authors contributed to the article and approved the submitted version.

## Conflict of Interest

The authors declare that the research was conducted in the absence of any commercial or financial relationships that could be construed as a potential conflict of interest.

## Publisher’s Note

All claims expressed in this article are solely those of the authors and do not necessarily represent those of their affiliated organizations, or those of the publisher, the editors and the reviewers. Any product that may be evaluated in this article, or claim that may be made by its manufacturer, is not guaranteed or endorsed by the publisher.

## Footnotes

1
http://gnomad.broadinstitute.org/

2
https://www.clinicalgenome.org/working-groups/sequence-variant-interpretation/

3
https://varnomen.hgvs.org/

4
http://swissmodel.expasy.org

5
http://genetics.bwh.harvard.edu/pph2

6
http://www.mutationtaster.org/

7
http://www.internationalgenome.org/

8
https://www.ncbi.nlm.nih.gov/pmc/articles/PMC4702902/

9
http://mendel.stanford.edu/SidowLab/downloads/gerp/

10
http://sift.bii.a-star.edu.sg/

11
http://compgen.cshl.edu/phast/

12
https://cadd.gs.washington.edu/

13
http://exac.broadinstitute.org/

## Abbreviations

HLDs: Hypomyelinating leukodystrophies
WES: Whole exome sequencing
*POLR1C*: RNA polymerase I and III subunit C
PMD: Pelizaeus–Merzbacher disease
PLP1: Protein proteolipid protein 1
ACMG/AMP: The American College of Medical Genetics and American College of Pathologists
vcf: Variant call format
OMIM: Online Mendelian Inheritance in Man.
